# Actinide bioimaging in tissues: Comparison of emulsion and solid track autoradiography techniques with the iQID camera

**DOI:** 10.1371/journal.pone.0186370

**Published:** 2017-10-12

**Authors:** Stephanie Lamart, Brian W. Miller, Anne Van der Meeren, Anissa Tazrart, Jaime F. Angulo, Nina M. Griffiths

**Affiliations:** 1 Laboratoire de RadioToxicologie, CEA, Université Paris-Saclay, Arpajon, France; 2 College of Optical Sciences, The University of Arizona, Tucson, Arizona, United States of America; FRANCE

## Abstract

This work presents a comparison of three autoradiography techniques for imaging biological samples contaminated with actinides: emulsion-based, plastic-based autoradiography and a quantitative digital technique, the iQID camera, based on the numerical analysis of light from a scintillator screen. In radiation toxicology it has been important to develop means of imaging actinide distribution in tissues as these radionuclides may be heterogeneously distributed within and between tissues after internal contamination. Actinide distribution determines which cells are exposed to alpha radiation and is thus potentially critical for assessing absorbed dose. The comparison was carried out by generating autoradiographs of the same biological samples contaminated with actinides with the three autoradiography techniques. These samples were cell preparations or tissue sections collected from animals contaminated with different physico-chemical forms of actinides. The autoradiograph characteristics and the performances of the techniques were evaluated and discussed mainly in terms of acquisition process, activity distribution patterns, spatial resolution and feasibility of activity quantification. The obtained autoradiographs presented similar actinide distribution at low magnification. Out of the three techniques, emulsion autoradiography is the only one to provide a highly-resolved image of the actinide distribution inherently superimposed on the biological sample. Emulsion autoradiography is hence best interpreted at higher magnifications. However, this technique is destructive for the biological sample. Both emulsion- and plastic-based autoradiography record alpha tracks and thus enabled the differentiation between ionized forms of actinides and oxide particles. This feature can help in the evaluation of decorporation therapy efficacy. The most recent technique, the iQID camera, presents several additional features: real-time imaging, separate imaging of alpha particles and gamma rays, and alpha activity quantification. The comparison of these three autoradiography techniques showed that they are complementary and the choice of the technique depends on the purpose of the imaging experiment.

## Introduction

Internal contamination with actinides can lead to long-term retention of these alpha-emitters in tissues [1]. Actinides can be localized in tissues at the site of entry (e.g., lungs after inhalation) or at a wound site, and following absorption to the blood, in systemic tissues that preferentially retain actinides (e.g., bone, liver). Contamination characteristics (route of entry, physico-chemical form of the contaminant, type of actinide) may influence the actinide distribution patterns within and between tissues. Alpha particles emitted by actinides with energies of the order of 5.5 MeV deposit energy within a range of about 40 μm in soft tissues. Therefore the radiation exposure of tissues and cells depends highly on the spatial distribution of the actinides in these biological structures. To correlate health effects or radiation dose to actinide distribution, it has been necessary in radiation toxicology to develop means to characterize the spatial distribution of actinides in tissues. Autoradiography techniques were developed to detect alpha emissions from tissue sections and hence visualize actinide tissue distributions.

One major technique based on principles of photography uses an emulsion of silver grains sensitive to charged particles and hence to alpha particles [2, 3]. With this technique actinides have been localized from the tissue scale [4] to the sub-cellular scale [5, 6]. Matsuoka *et al*. [4] observed the distribution of plutonium activity in liver, spleen and bone following administration of plutonium as a monomer (ion) or polymer (colloid) using whole-body autoradiography. After inhalation experiments with an aerosol of PuO_2_, Sanders *et al*. [5, 6] observed oxide particles of plutonium in tracheal, bronchial, and alveolar regions of rat lungs using scanning electron microscopy of autoradiographs. Furthermore, Sanders *et al*. related radiation dose from oxide particle aggregates deposited in rat lungs, to carcinogenic effects [7]. This emulsion-based technique was also used to assess the size of oxide particles [8, 9]. In addition, quantification of plutonium activity has been carried out in lungs of deceased nuclear workers [10].

Another important technique relies on an etchable plastic polymer, namely polyallyl diglycol carbonate. One of the forms of this plastic is known as CR-39 (Columbia Resin). These polymers belong to the family of Solid State Nuclear Track Detectors (SSNTD), [11, 12], which have broad applications in radiation measurements for identification and quantification purposes. Plastic-based autoradiography has been used to determine the size of plutonium oxide particles [13, 14]. It was also shown using CR-39 that the diethylenetriaminepentaacetic acid (DTPA), a decorporating agent, was efficient in removing soluble forms of Pu from rat lungs, but that oxide particles of Pu were still retained after DTPA treatment [15]. The activity retained in human lung tissues was also estimated with this technique [16]. In addition, radiation dosimetry based on plastic detectors has been applied especially for external neutron exposure [17, 18].

However, the quantification of activity from these autoradiography techniques remains generally a tedious task and activity calibration is difficult for autoradiographs of tissue sections contaminated with actinides. Indeed, quantification relies on visual grain counting, reflectance or absorbance measurements [19]. Furthermore, quantification becomes challenging when alpha tracks are superimposed on each other [20], which is likely to occur with insoluble actinides or aggregates.

To facilitate quantification of activity distribution in samples, new bioimaging techniques have been developed most recently: the *alpha camera* [21], the *α-Camera* [22], the *iQID camera* [23], which are based on a thin film detector and a digital camera; and the *Timepix* detector, which is based on an array of semi-conductors [24]. The alpha camera [21] is a low-spatial resolution (~0.6 mm) alpha imager that uses a position-sensitive photomultiplier tube to amplify scintillation light. The α-Camera [22] is an integrating digital autoradiography imager that does not detect alpha particles on an event-by-event basis but integrates all scintillation light to produce an image. These recent techniques enable quantification of the activity distribution in tissue sections. They have been increasingly applied for research in targeted alpha radiotherapy [25–28], with derivation of absorbed dose estimates [27]. In the frame of internal contamination with actinides, re-analysis of archival tissues is important to answer new questions that could not be addressed with techniques from the past [29, 30]. This was elegantly shown by Hare and co-workers, where laser ablation-inductively coupled plasma mass-spectrometry enabled generation of quantitative cartographies of plutonium, thorium and uranium isotopes in tissue sections from former nuclear workers [31].

The objective of this work was to compare an emulsion, a plastic detector, and a new autoradiography technique, the iQID camera [23], using each application for the same selected biological material contaminated with actinides. To highlight performances and drawbacks of the different techniques, a comparative analysis of the obtained autoradiographs and images was carried out mainly based on acquisition process, activity distribution patterns, spatial resolution and feasibility of activity quantification.

## Materials and methods

### Biological samples selection and preparation for autoradiography

Several biological materials, including tissues and cells, were selected from archival and more recent animal contamination experiments with actinides (Table 1). These experiments were conducted according to the European and French guidelines for ethical animal research, which are stated in the articles cited in Table 1 and corresponding to each biological sample used in the present study. Experiments included: (i) two routes of intake in an experimental rat model (inhalation and wound) and a cutaneous deposition on pig skin *ex-vivo*, (ii) two actinides of interest (americium, plutonium) and (iii) two physico-chemical forms of the contaminant (nitrate and oxide). More precisely, contaminants contained plutonium oxide [32], mixed oxide (MOX) of uranium and plutonium [33, 34], and americium nitrate [35]. Because Pu-241 contained in MOX decays into Am-241 with a physical half-life of 14 years, the MOX compound contains an increasing amount of americium oxide. Only the activity distribution of americium and plutonium was considered in this work, as uranium isotopes are mainly chemo toxic with specific activities from 10 to 10^8^ less than those of plutonium and americium isotopes.

**Table 1:** Examples of selected biological samples contaminated with actinides.

Sample identification	Sample type	Mode of contamination	Contaminant	Duration of the study	Section thickness (μm)	Reference
ASIE 16 11	Macrophage cells	Inhalation	PuO_2_	7 days	NA	[32]
IPAU 7054	Lung	Inhalation	MOX	3 months	5	[34]
IPAU 7053	Lung	Inhalation	MOX	3 months	30	[34]
MB 111 22	Muscle	Wound	MOX	140 days	10	[33]
CAT 154 C2	Skin	Cutaneous, ex-vivo	Am nitrate	24 h	10	[35]

MOX: mixed oxide of uranium, plutonium and americium. NA: not applicable.

Sample preparation for autoradiography depended mainly on the nature of the biological material (tissue or cells). Tissues were sampled from contaminated animals, fixed in formalin, dehydrated using increasing degrees of alcohol (ethanol, butanol, xylene) and paraffin embedded. Tissue sections were obtained by cutting the formalin-fixed paraffin embedded tissues (FFPE) using a microtome and placed on a microscope slide. A section thickness (5–30 μm) thinner than the alpha range was chosen so that emitted alpha particles could exit the section (Table 1). Actinides contained in the contaminants are all alpha emitters with similar energy, i.e. of around 5.5 MeV, and thus have similar ranges of about 40 μm in water and paraffin. A thin tissue section was also necessary for histological observations. Only for the emulsion-based autoradiography technique, the paraffin was removed from the tissue sections and sections were rehydrated using decreasing degrees of alcohol (from xylene to water). For this reason, the emulsion-based technique was applied after acquisitions with the other techniques.

In addition to tissue sections, samples of macrophage cells were obtained by broncho alveolar lavages of rats contaminated by inhalation of actinides. The cell suspension was centrifuged and cells were collected on microscope slides (cytospin technique).

FFPE whole tissues or tissue sections can be preserved for long periods of time and can be used several years after preparation. Hence a tissue bank has been generated over the years in the laboratory. On the other hand, after cyto-centrifugation, cells can be preserved at -20°C for years. As an example, the « ASIE 16 11 » sample had been generated 10 years before the acquisition date of the autoradiographs presented in this work.

### Alpha autoradiography using a nuclear track emulsion of silver halide crystals

All the following steps were carried out in a dark room to prevent the exposure of the silver grain emulsion to visible light and the subsequent fogging of the emulsion. The Kodak autoradiography emulsion type NTB (Stansted, UK) has silver halide crystals with a diameter of 0.4 μm before development. The emulsion, solid at room temperature, was heated in a water bath with a temperature of 43–45°C for one hour. Each microscope slide with a tissue section was immersed in the liquefied emulsion diluted with 50% of water. The emulsion layer was made as thin as possible by keeping the slide vertical allowing the heated emulsion on the slide to drip and thin by gravity flow to the base of the slide for a few seconds. The reverse side of the slide was wiped clean. The slide was placed in a light-tight slide box. The box was wrapped to protect from light and placed in the refrigerator at 2–8 C to solidify the emulsion for the duration of the exposure. The emulsion is thus in direct contact with the tissue section. Exposure times depended on the sample activity and activity spatial concentration. Serial sections and cell preparations were removed from the storage containers and developed as for photographic "film" to obtain optimal exposures.

The exposed slides were developed by immersion in Kodak Dektol Developer for 5 minutes. The slide was rinsed with acetic acid (2%) for 30 seconds to stop the reaction and then fixed using the Kodak Fixer for 10 minutes and rinsed using distilled water for 5 minutes. Sections were counter-stained with Harris’ hematoxylin-eosin and cover-slipped for analysis (S1 Text). For cells, the preparation was stained with Giemsa to highlight cytoplasm (colored in pink) and nuclei (in blue).

Autoradiographs were visualized using a Nikon Eclipse E400 optical microscope and autoradiography images were acquired with a camera (S1 Table). Plan fluorite objectives with a magnification of x2, x20 and x40 were used. Emulsion-based autoradiographs of biological samples were assessed by several operators.

### Alpha autoradiography using a plastic detector

Pieces of a plastic detector were placed on top of FFPE tissue sections or cells protected with a mylar foil with a thickness of 3.5 μm. The plastic detector was maintained in close contact to the sample using a weight during the exposure time. This work used the TASTRAK^™^ detector (TASL, Ltd, Bristol, UK) with a density of 1.30 g.cm^-3^, a thickness of 500 μm and a cut-off angle of 20° for alpha particles (http://www.tasl.co.uk/plastics.php [36]). Exposed pieces of TASTRAK^™^ were etched in a caustic solution (NaOH 6N) at 80°C for an hour, rinsed in water, dried out and wiped clean with alcohol. TASTRAK^™^ autoradiographs were visualized using the same Nikon Eclipse E400 optical microscope as for the emulsion-based autoradiographs (Objectives x2, x20, x60).

### Alpha and gamma autoradiography using the iQID camera

**Fig 1:**
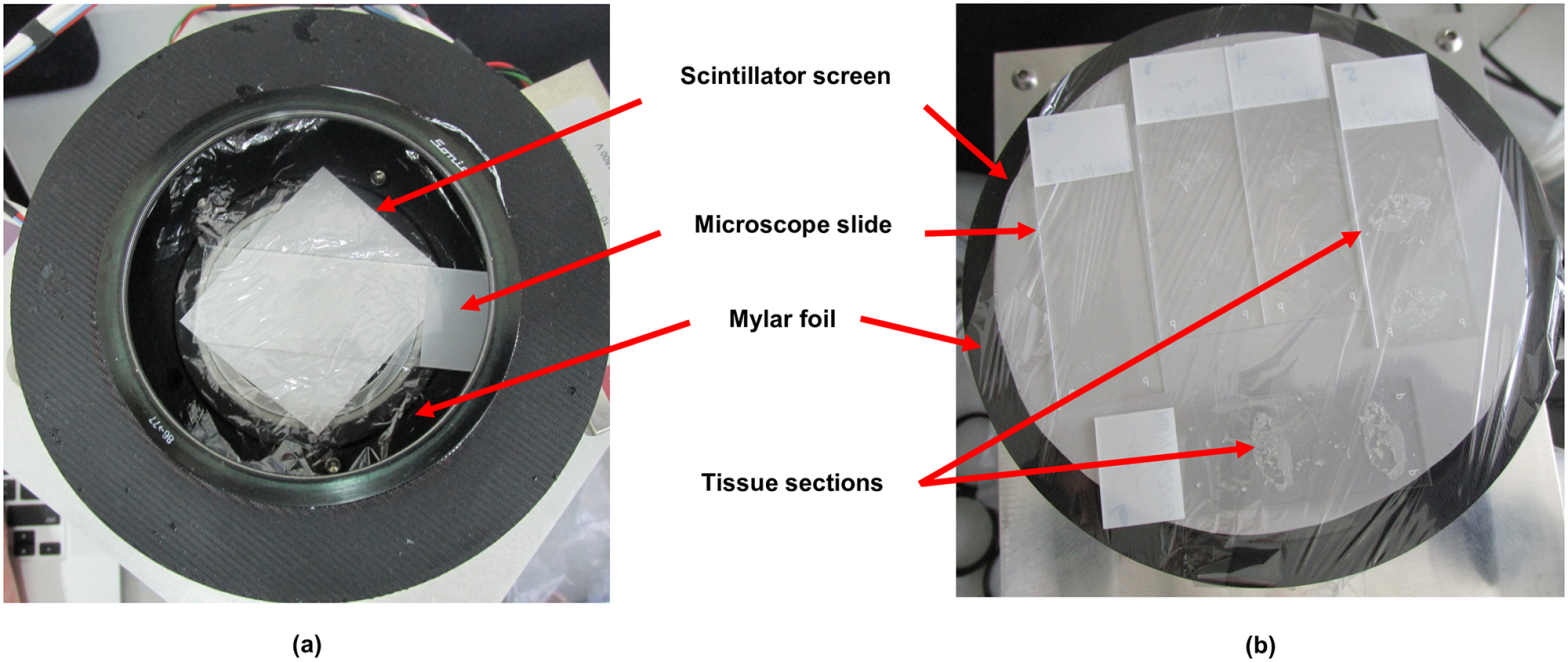
Top-view of microscope slides set-up on the two configurations of the iQID camera. (a) Camera with sensitive diameter of 40 mm and (b) with a fiber-optic tape that increased the sensitive diameter to 115 mm.

The iQID camera [23, 28] uses a thin film detector made of an inorganic solid scintillator [37], to convert alpha particle energy to a flash of light that is amplified and acquired by a charge-coupled device (CCD) camera. The technical aspects of the iQID camera have already been thoroughly described by Miller *et al*. (2015). Post-processing of the acquisitions enables recalculation of the position of the original alpha impact that created the flash of light in the scintillator, and hence reconstructs an image of the location of alpha particle emissions from the contaminated biological sample. Practically, the image of each alpha interaction corresponds to a contiguous region of pixels that is called an event cluster, e.g., 70 microns in diameter (FWHM). In real-time, individual alpha events are identified within each CCD camera frame and their interaction locations are estimated to the nearest pixel using a centroid calculation on cluster pixels. As interactions are localized, a counts image is generated where pixel values correspond to the number of alpha particles detected. Additionally, event clusters and their coordinates are also stored to disk where post-processing algorithms can be employed for final filtering and analysis, e.g., energy and particle type discrimination as well as removal of cosmic ray events. Two cameras were set-up to measure samples. Both cameras have a sensitive diameter of 40 mm, on which one to two slides could be placed (Fig 1a). In addition, a fiber-optic taper set-up on the camera served to increase the detector area to a diameter of 115 mm, which could accommodate five slides (Fig 1b and S1 Fig). Most of the acquisitions used a scintillator for alpha particle imaging composed of silver-activated zinc sulfide (ZnS:Ag) with a thickness of 100 μm. Some images were also acquired using a gadolinium oxysulfide (Gd_2_O_2_S) scintillator to image photons (e.g., 59 keV gamma rays from Am-241). The biological sample was placed in close contact with the thin film detector with only a mylar foil with a thickness of 3.5 μm in between for protection purposes. Samples were uniformly weighted using an aluminium plate for intimate contact across the field of the detector. A light-tight cover lid was placed over the samples during the acquisition to protect the camera from light exposure that would saturate and damage the camera’s image intensifier. Acquisition parameters were set depending on the initial sample activity detected by direct visualization of the alpha impacts with the camera. A Point Grey Research, Inc., 4.1 MP USB 3.0 CMOS camera (2048 × 2048 pixels) was used for these experiments and acquired images at a rate of 20 frames per second.

As stated above, a post-processing of each acquisition enables the generation of a 2D image of the distribution of alpha particles (or gamma rays) detected from the sample, where pixel values in the image correspond to the number of particles detected during the acquisition. In addition, computational codes were developed as ImageJ macros [38, 39] to automate, facilitate and make as reproducible as possible the activity and size calibration of each image as well as the assessment of sample activity. The macro for activity calibration takes as user inputs: (i) the iQID image and (ii) the acquisition time. In addition the user can select a region of interest for the activity calibration. The sample activity, A (Bq), was estimated assuming an efficiency of the camera of 100% in a solid angle of 2π sr using the total number of alphas in the sample and the acquisition time.

To determine reference values of alpha activity in tissue sections and cell samples, liquid scintillation counting was carried out on some samples from the same tissue or same cytospin preparation. Tissue sections or cells were wet-ashed using 2 HNO_3_ and H_2_O_2_ evaporated to dryness and resuspended in 2 HNO_3_. Sample activity was measured by liquid scintillation counting (Tricarb 2900, Perkin Elmer) using (Ultima Gold AB, Perkin Elmer, Courtaboeuf, France) liquid scintillant. An efficiency of 100% is assumed in these measurements.

## Results and discussion

### Autoradiographs characteristics

**Fig 2:**
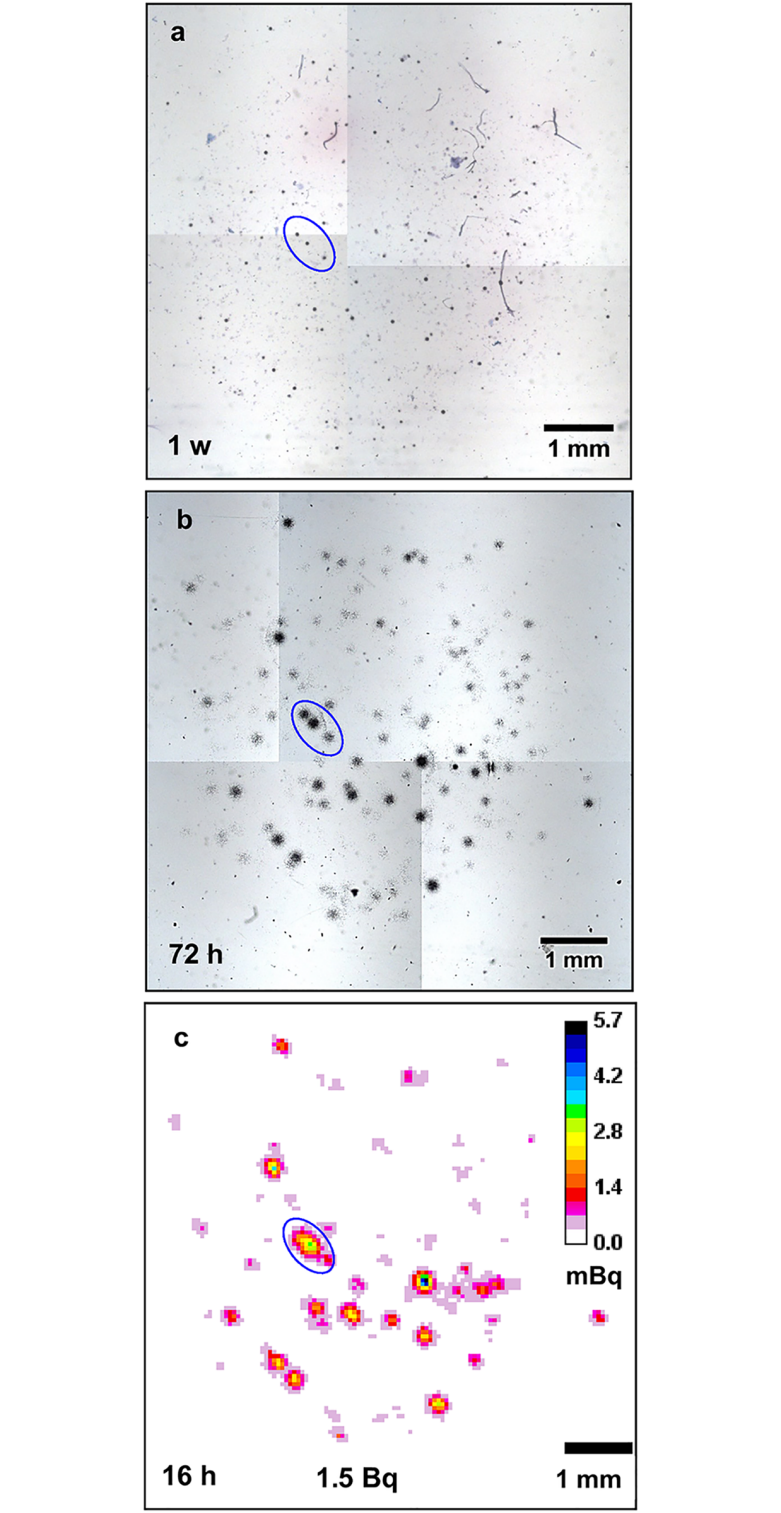
Autoradiographs from the same sample of rat lung macrophages after inhalation of PuO_2_. Nuclear track emulsion (a), plastic detector TASTRAK^™^ (b) and iQID autoradiographs (c). Exposure time is displayed on the lower left corner of each image. w: week; h: hours. Nuclear track emulsion technique was done last as it is a destructive endpoint. Three points of origin for appreciation of the spatial relationships and resolution of the tracks via each system are circled together in each frame. Emulsion- and plastic-based autoradiographs were observed with objective 2. The total alpha activity detected in the sample is displayed at the bottom of the iQID autoradiograph as estimated from this imaging technique.

The characteristics of autoradiographs from each technique will be successively discussed using a simple example of biological material (isolated cells, one cell type), composed of rat lung macrophages collected after PuO_2_ inhalation (Table 1, Sample identification: « ASIE 16 11 »; Fig 2). These cells were prepared with the cytospin technique.

#### Autoradiography using the nuclear track emulsion

**Fig 3:**
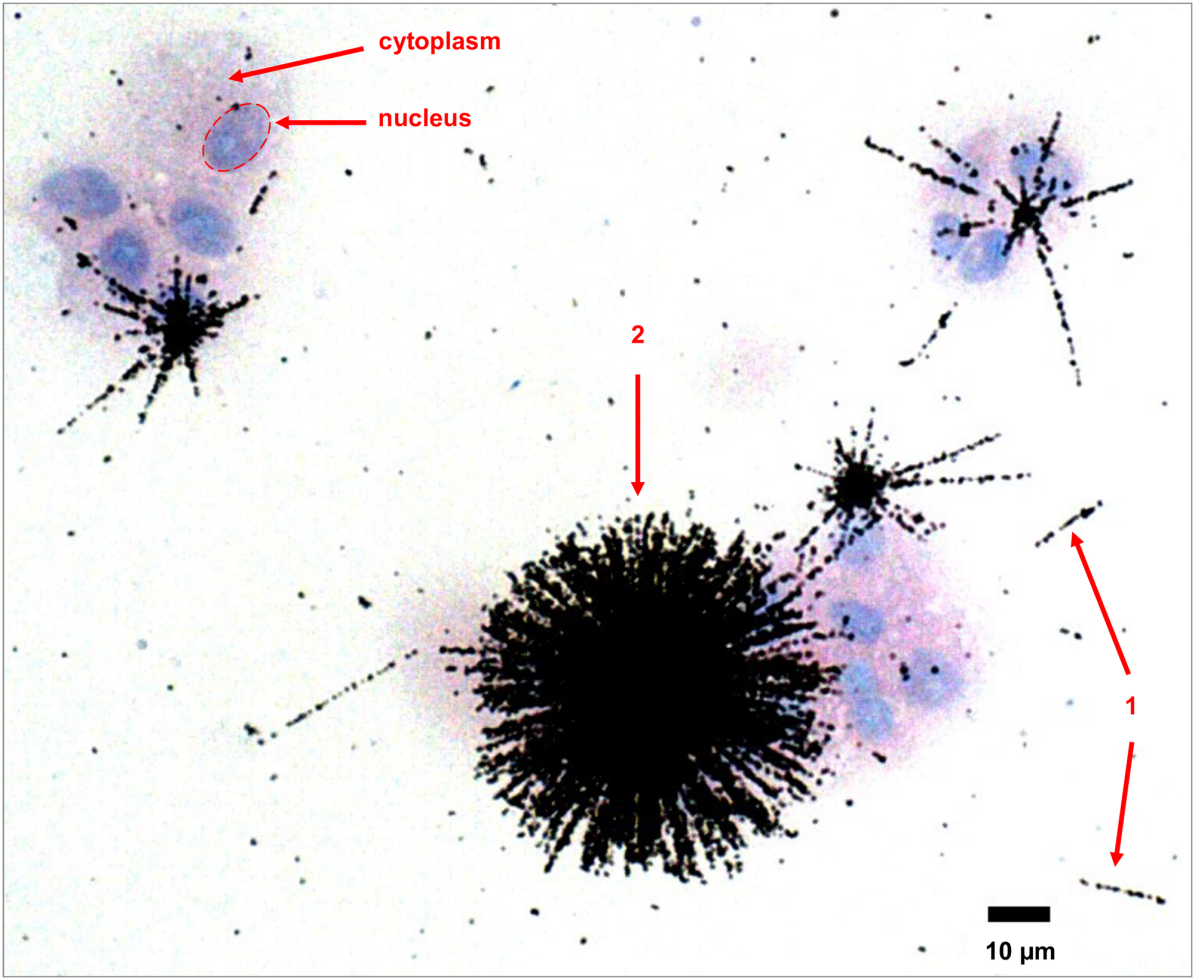
Stars and isolated alpha tracks in an emulsion autoradiograph of rat lung macrophages contaminated with PuO_2_. Samples were prepared with the cytospin technique. Giemsa staining was used. Cytoplasm in pink; nucleus in blue. Alpha tracks are black dotted straight lines. 1: isolated alpha tracks; 2: star. Objective used: 40.

In this technique, the silver halide crystals receive energy from the alpha particle along the particle trajectory in the emulsion. This energy deposition results in a materialization of the particle track as a trail of silver atoms, which are fixed and revealed by chemical photographic revelation. The actual particle track is thus recorded throughout the emulsion thickness. The resulting image consists in the two-dimensional projection of the alpha track that appears as a black dotted straight line (Fig 2a; and at higher magnification, Fig 3). Because the nuclear track emulsion was applied on the cell sample, this technique has the major advantage to provide an image of alpha track distribution directly superimposed on the cell preparation well visualized with cell staining. In case of contamination with oxide particles, a great number of alpha particles are emitted isotropically from the oxide particles, which results in the formation of stars (Fig 3, arrow 2). When the number of alpha tracks composing the star can be counted, an estimate of the oxide particle size can be derived [8]. In addition, a small fraction of the oxide particles can be dissolved and observed as soluble ionized form, which results in the generation of isolated tracks (Fig 3, arrow 1). These autoradiographs enabled visualization of the actinide location at the cellular scale depending on the quality of the emulsion, time of exposure, size of particles, microscope and camera quality. The track thickness, taken as the estimate of the image spatial resolution, was estimated to be in the order of 2 μm in the obtained images. This measure depends on the objectives of the microscope and the camera used to acquire the image. Good interpretation of emulsion-based autoradiographs requires observation at higher magnification as shown by the detail in Fig 3 (objective x40).

#### Autoradiography using an etchable plastic

**Fig 4:**
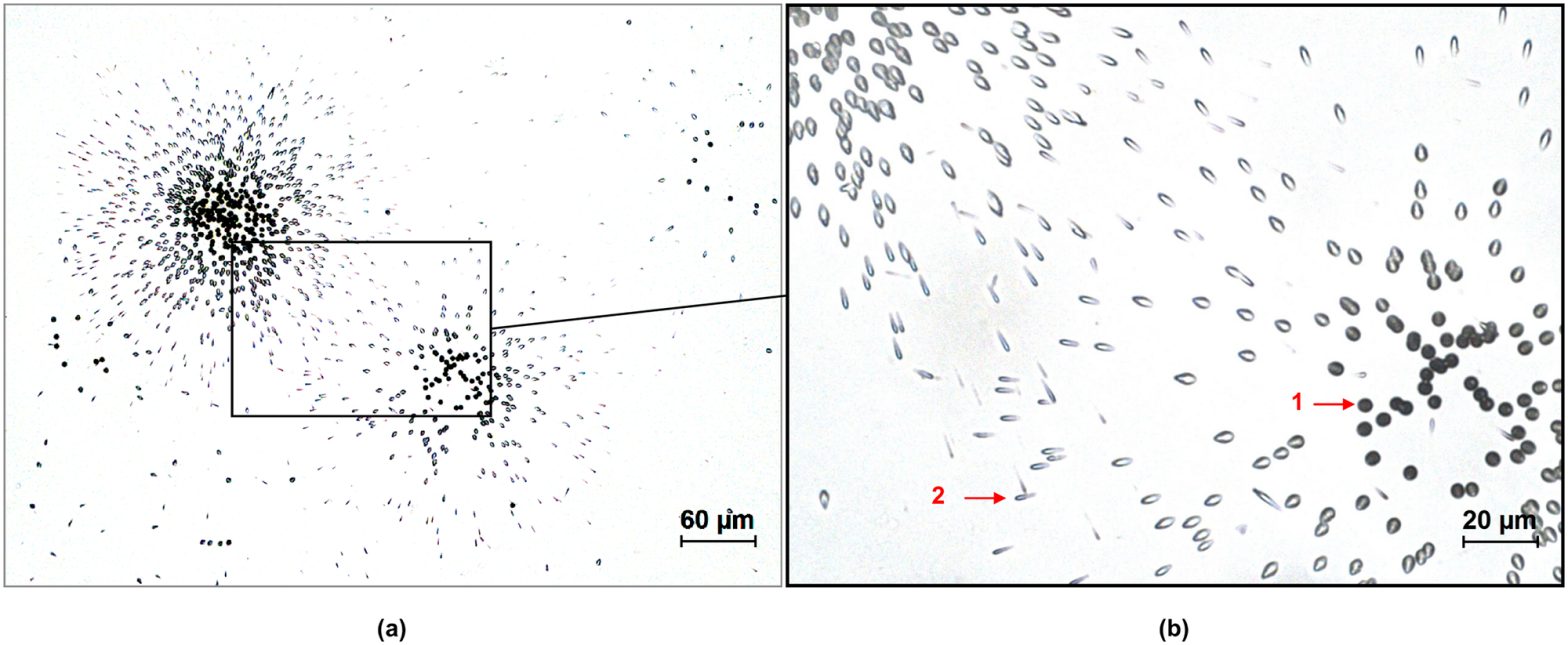
Alpha tracks from oxide particle in the plastic detector TASTRAK^™^ at two magnifications: objectives 20 (a) and 60 (b). Sample is from lung macrophages contaminated with PuO_2_. 1: orthogonal pit; 2: non-orthogonal pit.

The plastic detector is a solid-state nuclear track detector sensitive to charged nuclei including alpha particles [11]. When a charged particle intersects the material, it creates structural damage in the plastic that can be enlarged using caustic solution treatments that result in the formation of pits in the plastic. Therefore, like the emulsion technique, the plastic detector reveals alpha tracks (Fig 2b; and at higher magnifications Fig 4). These tracks resemble dots for particle incidences orthogonal to the detector (Fig 4b, arrow 1) and tears more or less stretched for non-orthogonal incidences (Fig 4b, arrow 2).

A pit diameter in the order of 4 μm was measured in the TASTRAK^™^ image from Fig 4b. Pit diameter is highly dependent of etching parameters and time [40, 41]. Hence, autoradiography with TASTRAK^™^ enabled the localization of alpha-emitter distribution within the field of the biological sample boundary. Formation of a star in the track detector shows the presence of an oxide particle, from which particle size can be estimated [14]. Oxide particles can thus be differentiated from ionized, i.e., soluble, forms of actinides, as for emulsion autoradiography.

#### Autoradiography with the iQID camera

The scintillator coupled to the iQID camera records the position of the interaction of alpha particles. This record enables reconstruction of the 2D locations of the alpha emitters in the biological samples (Fig 2c). For an iQID camera equipped with a 40-mm diameter detector area and an effective pixel size of 24.36 μm, the spatial resolution evaluated from the full width at half maximum of a line spread function was 32.3 μm for a 100-μm thick CdWO_4_ crystal and 46.6 μm for a thin layer (~25 μm) of ZnS:Ag phosphor that is affixed to a 250 μm thick plastic substrate. A thicker substrate increases lateral spread of scintillation light reaching the detector, which results in a lower spatial resolution due to higher uncertainty of the estimated interaction location.[28].

This device has the capacity of real-time imaging: alpha interactions with the scintillator were directly imaged and visualized on the computer screen at the time of acquisition (S1 File). This functionality facilitated the estimation of the necessary acquisition time and acquisition parameters, and also provided a preview of the 2D activity location in the sample.

As the iQID camera works as a counter, pixel values in the acquired images represent the actual number of detected alpha particles. The activity in the tissue section is estimated from the total number of alpha particles collected during the acquisition. Statistical uncertainty of activity estimation follows Poisson statistics (S2 Table).

### Application of the three techniques to tissues contaminated with actinides

**Fig 5:**
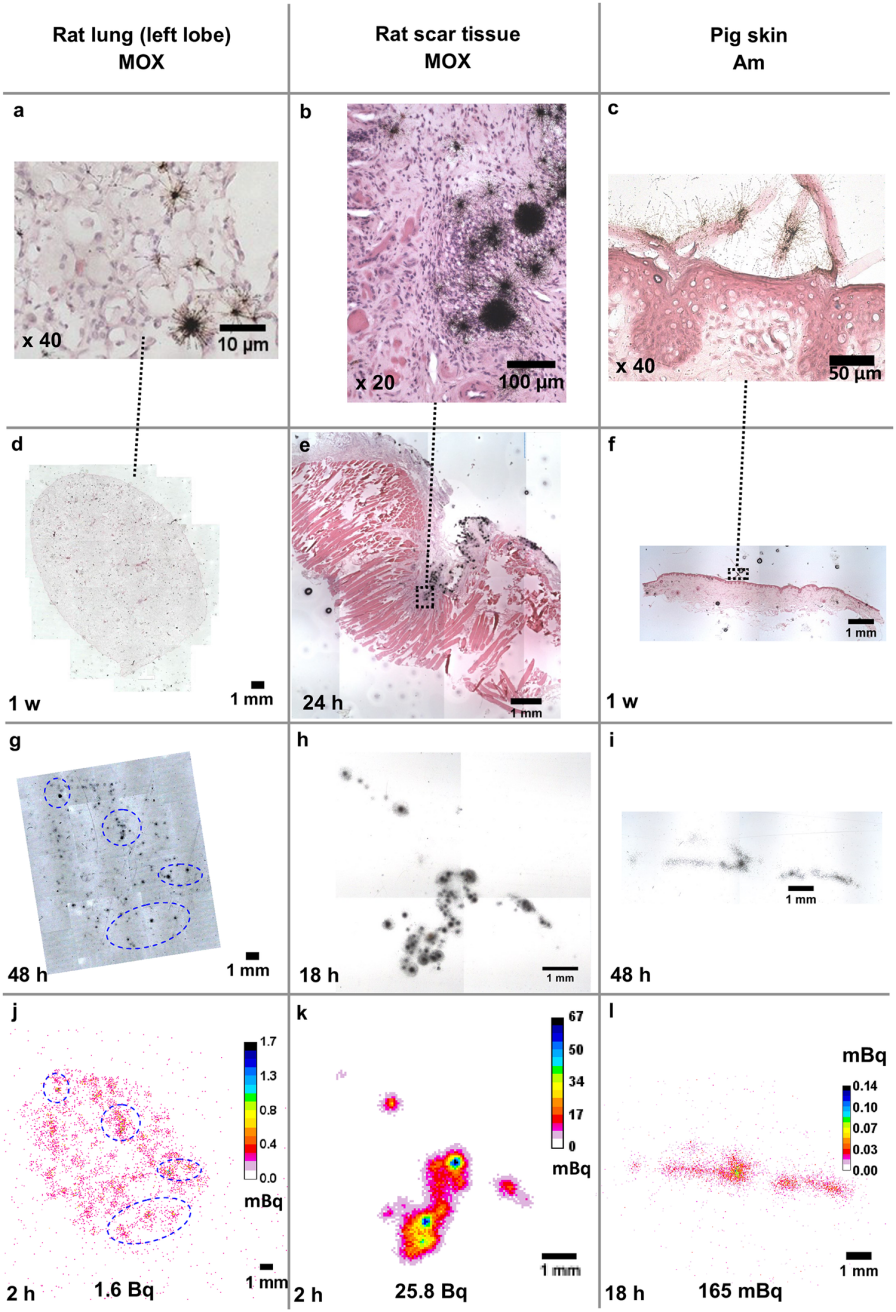
Actinide distribution in different animal tissue samples imaged by three autoradiography techniques. Nuclear track emulsion (a-f), plastic detector TASTRAK^™^ (g-i) and digital autoradiography with the iQID camera (j-l). MOX: mixed oxide of uranium, plutonium and americium. Exposure time is displayed on the lower left corner of each image. Emulsion- and plastic-based autoradiographs were observed with objective x2 (d-i). Blue ellipses were represented to facilitate the visualization of similar areas between plastic-based and iQID images for the lung section; the co-localization with the corresponding histological image remained difficult.

To compare performances and drawbacks of the three autoradiography techniques for contaminated tissues, these were applied on tissue sections from lung, muscle scar tissue and skin contaminated with actinides (Table 1, sample identification: « IPAU 7054 », « MB 111 22 », « CAT 154 C2 », respectively). The acquired autoradiographs enabled the evaluation of actinide distribution in these tissues at different scales (Fig 5). At low magnification, the autoradiographs presented similar distribution between techniques, especially in the scar tissue (Fig 5e, 5h and 5k).

The emulsion technique enabled the addition of a specific histological stain to identify a tissue structure or a cell type and the coincident overlay of detected alpha particles associated with the histological structures. The observation at high magnification of alpha tracks superimposed on the stained biological sample permits identification of the cells exposed to radiation within the tissue (Fig 5a). At low magnification (x2 objective), alpha tracks may be difficult to observe with the emulsion technique, especially for the lung and skin samples (Fig 5d and 5f).

**Fig 6:**
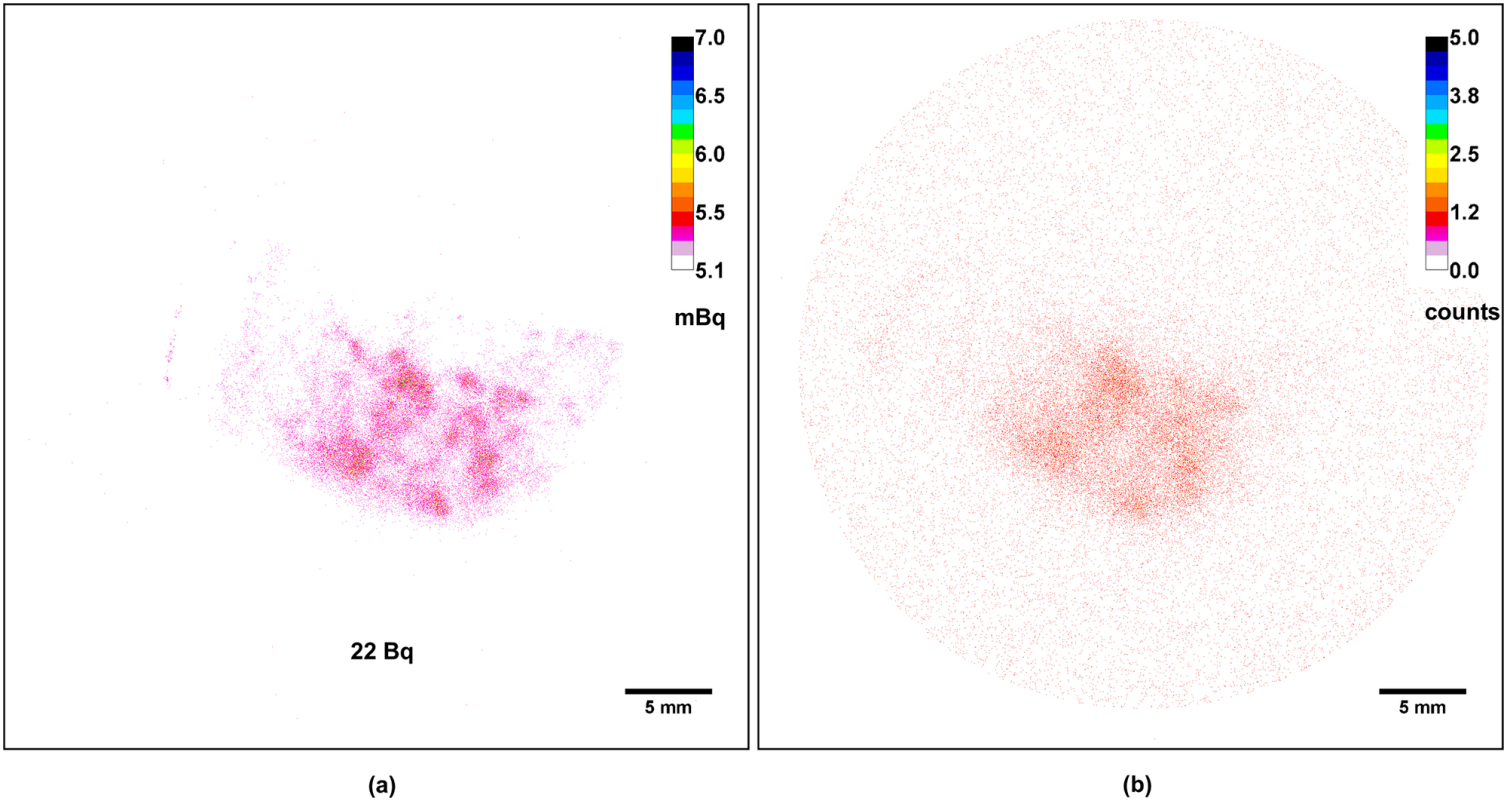
Comparison of the alpha (a) and gamma (b) autoradiographs of a rat lung tissue section contaminated with a mixed oxide of uranium, plutonium and americium (MOX). Acquisition time with the iQID camera was 2 h for both images. The total alpha activity in the sample is displayed at the bottom of the alpha autoradiograph.

The iQID camera permitted quantification of retained activity in the biological samples based on the total number of detected alpha particles and exposure time. As the detection efficiency of the technique was about 100%, activities as low as hundreds of mBq could be measured (e.g., total of 165 mBq in pig skin). Poisson statistics gave standard deviations representing 0.5% to 1.4% of the estimated activities (S2 Table). Activity estimation was comparable to activity measured by alpha spectrometry. For example, in a lung section close to the one used in Fig 6, 98 Bq were measured by liquid scintillation counting in a thickness of 60 μm, which is in the same order of magnitude as the estimated activity in the imaged lung section with a thickness of 30 μm: 22 Bq.

An interesting additional feature of the iQID camera relies on the possibility to discriminate between alpha particles from gamma-ray photons. While decay of Am-241 leads to alpha emission, a gamma ray at 59 keV may be subsequently emitted at a yield of 36%. An archival sample of lung tissue had been collected after contamination by inhalation with MOX (Sample identification: « IPAU 7053 » in Table 1). Ageing of this nuclear fuel resulted in the ingrowth of Am-241 [42, 43]. At the time of acquisition, 32% of Am-241 was expected to have been generated in activity from the initial MOX compound. This lung tissue sample contaminated with MOX was imaged using the two types of scintillators: (i) ZnS:Ag, which is sensitive to alpha particles only, and (ii) Gd_2_O_2_S, sensitive to gamma rays only (a protective plastic coating, thicker than the alpha range, enables stopping of alpha particles emitted from the sample) (Fig 6).

The image of alpha particles detected by the iQID camera showed that 3 months after lung contamination with MOX the actinides were heterogeneously distributed in the left pulmonary lobe (Fig 6a). The image of gamma rays presented a similar non-uniform distribution as compared with the image of alpha particles, but at a lower spatial resolution due to a thicker scintillator and to the nature of photon interactions with matter (Fig 6b). The background outside the tissue limit was also much greater in proportion to the signal as compared with the alpha image (1 x 10^−2^ counts per pixel versus 5 x 10^−5^ counts per pixel, respectively). Activity estimation from the gamma-ray imaging experiment could not be assessed as the imager was not calibrated for gamma-ray detection efficiency at the time of these measurements. Moreover, for one disintegration, i.e., one alpha emission, there is only a 0.36 probability of the 59.5 keV gamma emission. This observation in this particular sample enabled concluding that Am-241, as decay product of Pu-241, had the same distribution as Pu isotopes after lung contamination with MOX at the sub-tissue scale.

### Comparison of the three techniques

The application of the three techniques to the same biological samples enabled an intercomparison study (Table 2).

**Table 2:** Comparison of the three autoradiography techniques used to image actinide contaminated biosamples from various experimental conditions.

	Autoradiography technique		
	Nuclear track emulsion	Etchable plastic	iQID
Principle of alpha particle detection	Energy deposition by the alpha particle transforms silver ions to silver atoms	Ionization of the plastic	Excitation of atomic electrons of the thin film scintillator by the alpha particle
	Slowing down of the particle generates tracks	Slowing down of the particle generates tracks	Deexcitation through emission of scintillation light captured by the camera
Autoradiograph generation after particle detection	Photographic development	Chemical etching	Real-time imaging
			Immediate generation of the image
			Unrestricted dynamic range, i.e., no image saturation effects
Location of the alpha particle record	In the emulsion superimposed on the sample	In the plastic	Directly stored numerically
Main materials	Nuclear track emulsion	Etchable plastic	iQID camera
	Dark room	Caustic reagent	Personal Computer with Graphical Processor Units
	Electron/optical microscope, camera	Electron/optical microscope, camera	
Required expertise	Experimental (+++), biology	Experimental (+)	Imaging/Image analysis
Sample integrity	Destructive	Non-destructive	Non-destructive
Radiations	Charged nuclei including alpha particles on the same image	Charged nuclei including alpha particles on the same image	Alpha and beta particles, gamma rays
			Possibility to discriminate between alpha particles from gamma rays by scintillation signal and choice of scintillator
Image visualization	Electron/optical microscope	Electron/optical microscope	Computer screen
	Possibility to digitalize the image using a camera	Possibility to digitalize the image using a camera	
Image resolution	Track thickness in the order of 2 μm	Pit diameter in the order of 4 μm	FWHM of 32.3–46.6 μm from a line spread function
Particle size estimation	Possible	Possible	Not possible yet
Activity quantification	Not always feasible; Extremely tedious	Not always feasible; tedious	Real-time, direct and easy
Approximate cost	Kodak emulsion bottle: 1500 euros for 100 sections	2000 euros for 8 sheets of 50 cm * 33 cm	Infinite number of samples
	+ cost of microscope and camera	+ cost of microscope and camera	Camera from 6000–20000 euros

FWHM: full width at half maximum.

The techniques compared in this work rely on different principles and materials to generate autoradiographs. Regardless of the technique, the short range of alpha particles in air requires maintenance of close contact between the sample and the imager. It requires also generation of thin tissue sections to ensure that emitted alpha particles can exit the sample and be collected. Both the emulsion- and the plastic-based techniques show actual particle tracks, whereas the iQID camera detects alpha particles via scintillation. Although the three autoradiography techniques rely on different processes for image generation, the resulting images presented similarities at low magnification depending on the amount and spatial distribution of activity (Figs 4 and 5). This observation allows the validation of these imaging techniques for different samples contaminated with alpha-emitters. However, alpha tracks can be difficult to visualize at low magnification using the emulsion technique (Fig 5d and 5f).

Out of the three techniques, nuclear track emulsion autoradiography is the only one to provide an image of alpha tracks superimposed on the biological material, without the use of any additional image registration technique. This feature permits co-localization of actinide distribution with the cells. Although plastic-based or iQID images might be manually aligned to the histological image, the accuracy of this superimposition depends on the sample and can be challenging to achieve exact spatial co-ordination.

Image resolution is intrinsically linked to the process of particle recording, treatment and means of autoradiograph visualization. With regard to nuclear track emulsion, the interaction of alpha particle with silver halide crystals leads to the formation of silver atoms [3]. In the case of plastic detectors, ionization by alpha particles induces structural damages. The development steps for nuclear track emulsion and immersion of the plastic in caustic solution results in the enlargement of the original track size in both techniques. On the other hand, the processing of iQID images that reconstruct alpha particle position from the flash of light generated in the scintillator, on event-by-event basis, enables the improvement of the resolution by reducing the size of the original event created by the alpha particle interaction with the detector. However, image resolution remains at least 10 times better with the emulsion than with the iQID camera. As a result, the emulsion-based autoradiography is the method of choice for observations at cellular or sub-cellular scale, whereas the iQID camera is best suited for observations at higher scales, for example, for small clusters of cells or for a whole tissue section, i.e., lung lobe (Fig 5).

Both the plastic-based technique and the iQID camera preserve the integrity of the sample, whereas the use of a nuclear track emulsion does not. The sample cannot be reused for another alpha imaging experiment because the autoradiograph composed of silver atoms is irreversibly fixed on the sample. Therefore, it is important with the emulsion to evaluate properly the time of exposure at the first attempt to prevent generating under- or over-exposed samples. However serial sections (≤ 5 μm) can still be used for different times of exposure or different techniques if necessary. On the contrary, acquisitions can be repeated on the same sample as needed with the plastic or iQID camera.

Unlike with the emulsion- and plastic-based techniques, the image generation with the iQID camera is direct, which enables imaging of alpha particles in real-time. This feature is useful to obtain a rapid overview of the activity distribution in the sample, whereas the other two techniques necessitate longer exposures and chemical post-processing before achieving such an evaluation. The iQID camera could thus guide the estimation of sample exposure time, in particular for the emulsion autoradiography, which cannot be repeated on the same sample. An additional feature of the iQID camera as compared with classical techniques relies on the possibility to image separately alpha and gamma emissions, which is impossible with nuclear track emulsion or plastic detector. Application of this feature to contaminated samples could facilitate differential localization of a mixed alpha/gamma contaminant (e.g., Pu-238 from Am-241). While the re-use of archival tissues is becoming important in the frame of the 3Rs of ethical animal research (aka, as Replacement, Reduction, Refinement) [44], these functionalities could facilitate and make faster the exploration of sections from archival tissues from animal studies or indeed human studies [45].

Emulsion- and plastic-based images may not permit estimates of activity when tracks overlap or when track distribution is too heterogeneous as in the autoradiographs obtained in this work (Figs 2, 3 and 5). Even if these requirements are met, activity estimation remains a cumbersome process [10, 46]. Furthermore, multiple exposures, which are possible only with the plastic detector, may be needed to achieve good analyses. Relative or absolute quantification from nuclear track emulsion-based images have been based on visual counting of grain or tracks and on the measurement of the optical density. Contrary to the emulsion and plastic techniques, activity quantification is direct and easy from the iQID detector because it functions as an imager and radiation counter. This unique feature is essential for radiation dosimetry at the sub-tissue scale. Three-dimensional radiation dose has been estimated from iQID images of tissue sections and simulation of localized irradiation for application in targeted radiation therapy [27]. This method could be applied to reconstruct the radiation dose to tissues contaminated with actinides. Activity quantification and dose estimates could be used to assess the correlations between health effects and activity or radiation dose. The efficacy of decorporation therapy could be simultaneously imaged and quantified experimentally from a tissue sample. Furthermore, actinide distribution could be visualized and quantified on a biopsy from a contaminated person, for example from the excised tissues after wound contamination. In addition, given the possibility to identify both alpha and gamma emissions on a tissue sample this technique could also have a use in nuclear forensics.

## Conclusion

This work compared a new digital autoradiography technique with emulsion- and plastic-based techniques for application to biological material contaminated with actinides. Samples of different nature (tissue or cell), species (rat or pig) and contaminated with various forms of actinides (e.g., plutonium oxide, americium citrate) were selected. Autoradiographs were successively generated using each technique on the same selected samples. The comparison was mainly based on acquisition process, activity distribution patterns, spatial resolution and feasibility of activity quantification.

Autoradiography with nuclear track emulsion is the method of choice for localization of alpha-emitters at the cellular scale. It provides highly-resolved track images superimposed on the stained sample. As such, it is particularly useful to identify target cells.

The plastic detector is a rapid technique to characterize the alpha-emitter distribution, based on observation of track patterns from ionized forms of actinides versus oxide particles. It allows repeated exposures of the same sample to facilitate analyses.

The iQID camera has several advantages over these techniques: (i) real-time imaging, (ii) direct image generation and activity quantification, (iii) differentiation of alpha and gamma radiations. It provides activity distribution at the sub-tissue scale. With the latter approach, alpha-emitter distribution and activity estimates may be used for radiation dose estimation within tissues. Moreover, the iQID camera could be used to screen rapidly a large amount of contaminated biological samples from tissue banks and as such facilitate re-use of animal data. Finally, the three autoradiography techniques compared in this work are complementary and the choice of one technique over the others should be done depending on the purpose of the autoradiography experiment.

## Supporting information

S1 File Real-time imaging of a source with the iQID camera. The source contained Np-237, Am-241 and Cm-244 with activities of 245 Bq, 150 Bq and 49 Bq, respectively at the time of acquisition.
(AVI)

S1 Table Characteristics of the camera used to image autoradiographs. (XLSX)

S2 Table Estimation of activity in contaminated samples from iQID autoradiographs. (XLSX)

S1 Text Protocols for biological sample staining used in this work. (DOCX)

S1 Fig 3D drawing of the fiber optic taper magnifier. The scintillation light is transported by the fibers and concentrated on the 40 mm field of view. The “scintillation” image obtained on the 115-diameter base is reduced to an image with a diameter of 40 mm.
(PDF)
