# Supplementary material for: Actinide bioimaging in tissues: Comparison of emulsion and solid track autoradiography techniques with the iQID camera
Source: PLoS One. 2017 Oct 12;12(10):e0186370. doi: 10.1371/journal.pone.0186370 (PMC5638496; doi:10.1371/journal.pone.0186370)
Supplement: S1 Text — (DOCX) [file pone.0186370.s004.docx]

**S1 Text. Protocols for biological sample staining used in this work.**

**Haematoxylin-eosin staining**

1. Deparaffinisation of sections through series of alcohols to water
2. Stain with Harris-Haematoxylin solution (Labord, VWR International, Lille France) – 5 min
3. Rinse thoroughly with water – 5 min
4. Blue in 1 % Lithium carbonate (Sigma Aldrich, St Quentin Fallavier, France) – 30 seconds
5. Rinse thoroughly with water – 5 min
6. Differentiate in acid (HCl 1% v/v) /alcohol (70% v/v) – 10 to 30 seconds
7. Rinse thoroughly with water – 5 min
8. Counterstain with Eosin solution (1% wt/v); (Labord, VWR International, Lille France) – 5 min
9. Rinse thoroughly with water – 5 min
10. Allow to dry and mount (Eukitt, Labonord, VWR International, Lille, France) and coverslip

**May Grünwald Giemsa staining**

1. Stain with May Grünwald-Giemsa solution for 1-2 min
2. Transfer the slide without washing in May Grünwald solution diluted with an equal volume of phosphate buffer saline (PBS)
3. Rinse the slide in PBS
4. Place the slide for 15 min into Giemsa stain diluted with 7 volumes of PBS
5. Rinse thoroughly with distilled water
6. Allow to dry and mount (Eukitt, Labonord, VWR International, Lille, France) and coverslip
