# Supplementary material for: Actinide bioimaging in tissues: Comparison of emulsion and solid track autoradiography techniques with the iQID camera
Source: PLoS One. 2017 Oct 12;12(10):e0186370. doi: 10.1371/journal.pone.0186370 (PMC5638496; doi:10.1371/journal.pone.0186370)
Supplement: S1 Fig — The scintillation light is transported by the fibers and concentrated on the 40 mm field of view. The “scintillation” image obtained on the 115-diameter base is reduced to an image with a diameter of 40 mm. (PDF) [file pone.0186370.s005.pdf]

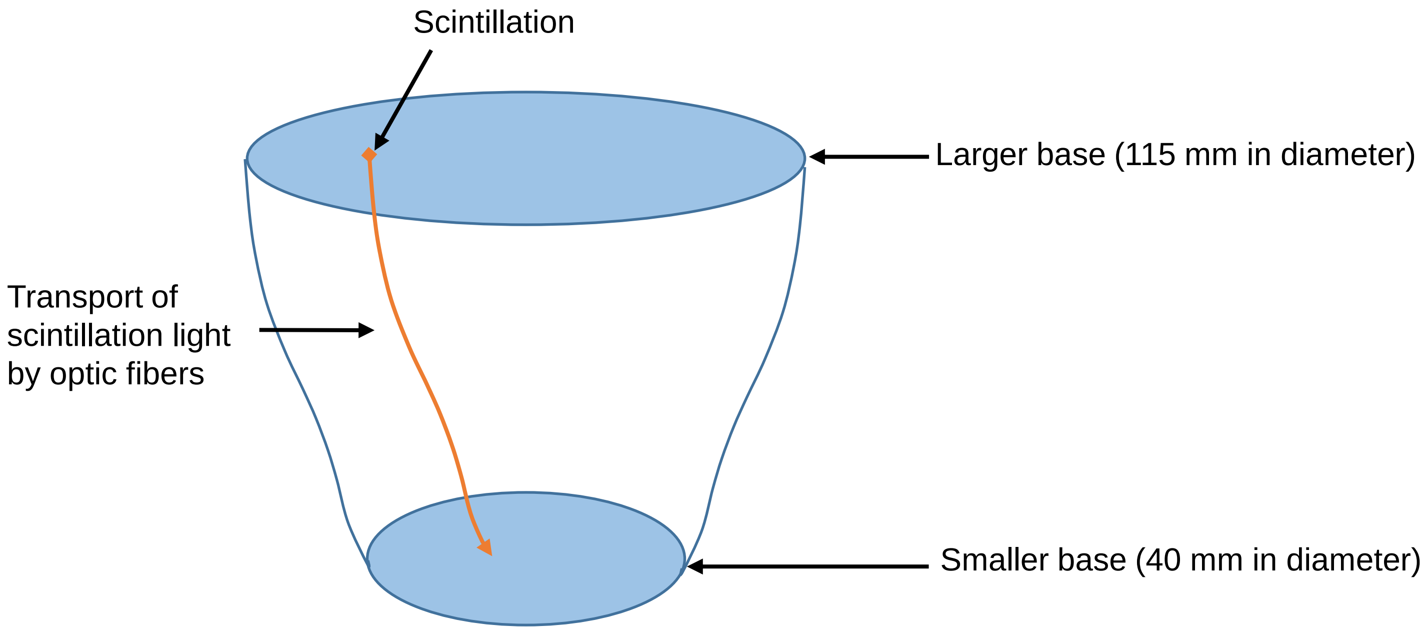

S1 Fig: 3D drawing of the fiber optic taper magnifier. The scintillation light is transported by the fibers and concentrated on the 40 mm field of view. The “scintillation” image obtained on the 115-diameter base is reduced to an image with a diameter of 40 mm.
